# Barriers to Immunosuppressant Medication Adherence in Thoracic Transplant Recipients: Initial Findings

**DOI:** 10.3390/ijerph22071090

**Published:** 2025-07-08

**Authors:** Sparkle Springfield-Trice, Grishma Reddy, Cara Joyce, Benito M. Garcia, Palak Shah, Sean Agbor-Enoh, Hannah Valantine

**Affiliations:** 1 Department of Public Health Sciences, Parkinson School of Health Sciences and Public Health, Loyola University Chicago, Maywood, IL 60153, USA; greddy@luc.edu; 2 Department of Medicine, Stritch School of Medicine, Loyola University Chicago, 2160 S 1st Ave, Maywood, IL 60153, USA; cjoyce6@luc.edu; 3 Department of Surgery, School of Medicine, University of Minnesota, Minneapolis, MN 55455, USA; garc1054@umn.edu; 4 Inova Schar Heart and Vascular, Falls Church, VA 22042, USA; palak.shah@inova.org; 5 School of Medicine, Johns Hopkins University, Baltimore, MD 21205, USA; sean.agbor-enoh@nih.gov; 6 School of Medicine, Stanford University, Stanford, CA 94305, USA; hvalantine@stanford.edu

**Keywords:** medication adherence, heart, lung, thoracic, transplant

## Abstract

Although transplantation remains the gold-standard treatment for patients with end-organ failure, lifelong adherence to immunosuppressant medication is required to prevent rejection, graft failure, and mortality. Given the increase in thoracic organ transplantation, it is crucial to better understand the associated barriers to treatment. Examining sociodemographic, transplant, healthcare access, post-transplant treatment, and patient-related psychosocial factors may help to elucidate treatment barriers that have not been previously considered in the existing literature. This single-site cross-sectional study surveyed 65 thoracic (heart and lung) transplant recipients (mean age: 62 years; 76.2% male; 72.3% White, and 21.5% Black) via phone interviews. Immunosuppressant nonadherence was found in 49.2% of participants (46.9% heart, 51.5% lung). In a four-week period, 20% of participants missed at least one dose, 40% did not take their medications on time, and 1% stopped completely. Significant correlates of nonadherence included poorer diet quality, fewer comorbidities, and maladaptive coping responses to perceived discrimination. This preliminary study highlights the importance of considering the social determinants of health—particularly post-transplant treatment and psychosocial patient-related factors—to inform post-transplant care. Addressing such variables may improve medication adherence and, subsequently, overall health outcomes. Further research with larger samples is needed to better understand the associated correlates and inform effective interventions for enhanced medication adherence.

## 1. Introduction

While transplantation is the gold-standard treatment for patients with end-stage organ failure, it necessitates lifelong immunosuppressant use for optimal outcomes [1]. In 2023, the Organ Procurement and Transplantation Network recorded new annual highs for liver, kidney, heart, and lung transplants. Specifically, the number of lung transplants surpassed 3000 for the first time, reaching a total of 3026. Additionally, all-time volume records were also set for heart transplants, totaling 4545 [2]. Given the increase in heart and lung (thoracic) transplants, research focused on post-transplant care is needed in this population [1,3,4]. This includes investigating the barriers to immunosuppressant medication adherence, as complete adherence is essential to post-transplant survival [5,6,7,8,9,10,11].

Medication adherence is the strongest modifiable factor in reducing infection, graft failure, and mortality in transplant recipients [5,8,12]. Following treatment guidelines is particularly crucial in the first-year post-transplant, where missing even one dose can disrupt therapeutic levels, increasing the risk of rejection and poor outcomes [13,14,15,16,17,18]. Yet, nonadherence remains a significant issue in transplant recipients [19,20,21]. Consequently, understanding the complex nature of non-adherence behaviors and the psychosocial environments in which they occur, is a pressing public health concern.

The World Health Organization categorizes predictive factors for medication adherence into five dimensions [22]: sociodemographics [15,18,23], transplant-related [18], healthcare access [24], post-transplant treatment [25], and patient-related psychosocial [18,21,26,27]. Most of the studies in this context have focused on recipients of kidney and liver transplants; therefore, the findings may not apply to thoracic recipients [28,29,30]. Available evidence suggests thoracic transplant recipients also experience a significant amount of medication nonadherence [24,31]. For instance, in an international cross-sectional study, Denhaerynck and colleagues (2018) found that heart transplant recipients reported a 34.1% nonadherence rate [24]. Similarly, Drick and team (2018) reported a 27.4% nonadherence rate among recipients of lung transplants [31]. Despite this evidence, few studies have examined adherence and its social predictors in thoracic transplant recipients, specifically.

Barriers to medication in thoracic transplant recipients occur on multiple levels. These include smoking, negative attitudes towards immunosuppressants, relational factors (such as having caregiving support), and healthcare policy factors (such as out-of-pocket costs and medication pick-up) [24]. For instance, recipients who smoked had twice the odds of confronting barriers compared to non-smokers [12,24], and those with negative attitudes towards immunosuppressants were over 11 times more likely to face barriers [12,19]. Most of the existing literature does not go beyond examining these traditional sociodemographic, lifestyle, and psychological factors. As such, they do not fully explain the effects of medication nonadherence and identity-based disparities in transplant outcomes, which necessitates an interdisciplinary approach [32].

Broadening patient-level psychosocial factors to include social determinants of health, such as stress and coping mechanisms, is timely and essential. In a recent study, Chan et al. (2022) called on national data sources focused on transplant recipients to include more information on social determinants, including perceived discrimination, which is rarely assessed in transplant databases [33]. A systematic review by Korb-Savoldelli and colleagues (2010) highlighted the lack of patient perspectives and the relationship between recipients and healthcare providers in studies on thoracic transplants [19]. This includes information on mental (e.g., perceived stress) and physical (e.g., diet quality) health outcomes [34]. For instance, discrimination has been linked to barriers to medication adherence among Black individuals with hypertension [33]. Additionally, patient-related factors (including perceived stressors such as discrimination [14]) and psychological assets (such as a patient’s resilience [35]) that could impact adherence to immunosuppressants are predominantly understudied in the existing literature [18].

To address these gaps in the literature, the present study aims to examine factors affecting medication adherence in thoracic transplant recipients using the five dimensions of adherence (sociodemographics, transplant-related, healthcare access, post-transplant treatment, and patient-related psychosocial factors). We expand patient-related psychosocial factors to include mental and physical health predictors that are relevant to the social determinants of health, such as perceived discrimination. We hypothesize that factors within all five dimensions significantly correlate with immunosuppressant nonadherence. Broadly, the purpose of our research is to identify contextual barriers to medication adherence in thoracic transplant recipients, enabling the development of effective strategies to improve adherence.

## 2. Materials and Methods

### 2.1. Study Design and Population

This ancillary study is part of the Genomic Research Alliance for Transplantation (GRAfT, NCT#02423070) study. Detailed information regarding the GRAfT study design has been published elsewhere [36]. At Loyola University Chicago (LUC), we conducted a cross-sectional survey via phone interviews with thoracic transplant recipients enrolled at a single GRAfT study site (Inova Schar Heart and Vascular). Data collection took place from May to June of 2021.

**Figure 1:**
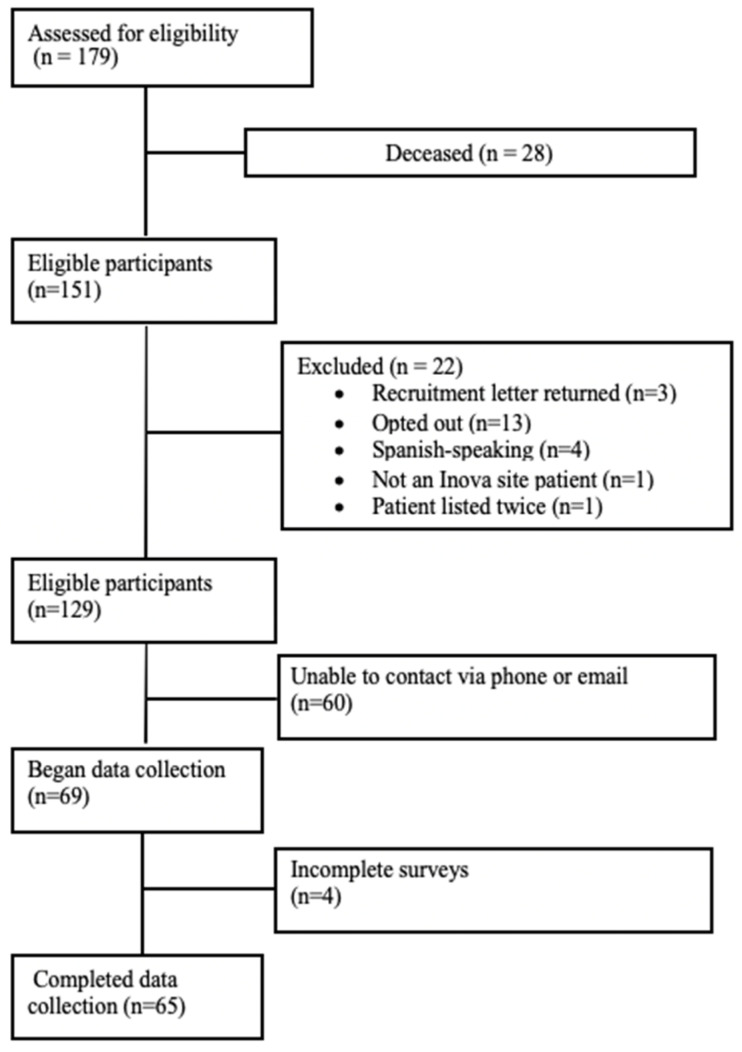
Flowchart of participant inclusion and exclusion criteria.

Eligible participants included adult recipients (≥18 years of age) who were English-speaking, approximately four years post-heart and/or lung transplant, and enrolled in the GRAfT study. Figure 1 illustrates the patient enrollment and exclusion process during the recruitment phase. Out of 129 eligible recipients who received a thoracic transplant, 65 completed the surveys and provided self-reported data, resulting in a sizable participant response rate of 50.4%. Nonresponse bias was mitigated by contacting each eligible participant up to five times using multiple communication modes.

### 2.2. Data Collection

After receiving approval from both the external WCG Institutional Review Board and Loyola’s internal Institutional Review Board, the GRAfT consortium provided Loyola staff with the contact information of eligible participants, including their full names, phone numbers, emails, and residential addresses. All participants were screened and consented to participate before the survey was administered. Loyola staff, comprising undergraduate and graduate public health students, conducted assessments via phone using a patient-centered, nonjudgmental, and nonthreatening approach. GRAfT researchers and staff reviewed all measures to assess their significance in evaluating the social determinants that influence disparities in thoracic transplant outcomes. All surveys were recorded on paper and cross-checked by the research team for quality control before being entered into the RedCap database. Data management was supported by the Clinical Research Office at Loyola University Chicago.

### 2.3. Primary Outcome: Basel Assessment of Adherence with Immunosuppressive Medication Scale (BAASIS)

The BAASIS© is a validated self-report instrument that assesses the primary outcome measure—namely, medication nonadherence—in the past month [37,38]. In particular, the BAASIS is a medication adherence scale [31,39,40]. Through validation in heart and lung recipients, the BAASIS was designed specifically to assess the adherence of transplant recipients and other chronically ill recipients to immunosuppressive medications, focusing on initiation, implementation, and persistence.

BAASIS includes four dimensions, with items focused on (1) taking adherence (missed doses), (2) drug holidays (skipping two or more doses), (3) timing adherence (doses taken > ± two hours), and (4) dose alteration (higher/smaller doses in the last month or stopping medication completely within last year). In accordance with the BAASIS scoring criteria, participants were classified as nonadherent if they responded “yes” to any item.

### 2.4. Exposure Measures: Five Dimensions of Adherence

**Figure 2:**
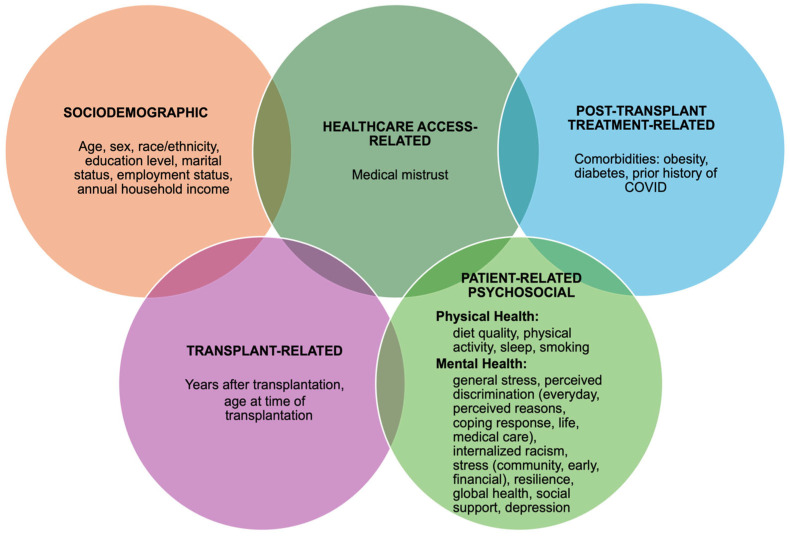
Five dimensions of factors contributing to immunosuppressant nonadherence.

Exposure variables were organized into five dimensions of adherence: sociodemographic factors, transplant-related factors, healthcare access-related factors, post-transplant treatment-related factors, and patient-related psychosocial factors. The variables are listed by dimension in Figure 2, and all exposure variables are briefly described below. Validated and reliable scales were used wherever possible. Detailed information of each measure can be found in Table A1 (Appendix A).

Sociodemographic variables included age, sex, race/ethnicity, education level, marital status, employment status, and annual household income. For transplant-related factors, we recorded the number of years since transplantation and the age at which the transplant occurred. Medical mistrust was evaluated as part of the healthcare access-related factors [41]. The post-transplant treatment-related dimension focused on medical comorbidities, specifically obesity, diabetes, and a history of COVID-19.

The psychosocial variables in the fifth dimension were divided into physical and mental health outcomes. Physical Health Outcomes: Diet quality was measured using the WELL Diet Score (0–120), with higher scores indicating better dietary habits [42]. Physical activity was assessed through the Godin Leisure-Time Exercise Questionnaire, with higher scores reflecting greater levels of activity [43]. Sleep was considered adequate if participants reported getting 7 to 9 h per night. Smoking status was determined according to whether participants identified as current smokers.

Mental Health Outcomes: Perceived stress was measured using the Perceived Stress Scale (PSS-4) (0–16), with higher scores indicating more stress [44]. Perceived discrimination was assessed using the Everyday Discrimination Scale [45]. The Coping Response to Unfair Treatment measure was used to assess the participants’ strategies for dealing with unfair treatment [46,47,48]. Major Discrimination Events evaluated significant experiences of discrimination [49,50]. Discrimination in Healthcare was measured using a single item assessing perceived discrimination in healthcare settings [51]. Internalized Racism was assessed using an abbreviated version of the Internalized Racism and Oppression Scale (IROS 5-item scale) (1–5), where higher scores indicate greater internalized racism [52]. Community Stress was evaluated using a five-item subscale for community violence, with higher scores indicating more stress [53]. Early Stress was measured using the Adverse Childhood Experiences Scale (0–8), with higher scores reflecting more adverse experiences [54,55]. Financial Stress was assessed through two questions measuring strain (1–5), with higher scores indicating greater stress [56]. Resilience was measured using a brief resilience scale (1–5) to assess self-reported ability to bounce back from stress; higher scores signify greater resilience [57]. Global Health was assessed using the PROMIS scale, which evaluates health across five domains, with higher scores indicating better functioning [58,59]. Social Support was measured using the SIMSS, which categorizes the number of support sources into low, moderate, and high levels [60]. The Patient Health Questionnaire-2 (PHQ-2) scores range from 0 to 6, with scores of 2 or higher suggesting the presence of a depressive disorder [61,62,63,64].

### 2.5. Statistical Analysis

To assess medication adherence in our sample, participants were described as adherent or nonadherent based on their BAASIS score. The results were also further stratified based on organ type (heart or lung). Continuous variables are described using means and standard deviations, while medians and interquartile ranges are used for ordinal variables. Categorical variables are described with frequencies and percentages.

To examine the sociodemographic, transplant-related, healthcare-access-related, post-transplant treatment, and patient-related psychosocial factors influencing medication adherence in thoracic transplant recipients, two-sided two-sample t-tests and Wilcoxon rank sum tests were conducted to identify differences in continuous measures between the adherent and nonadherent groups in our entire sample of thoracic transplant recipients. Chi-squared and Fisher’s exact tests were used to examine differences in categorical variables. *p*-values <0.05 were considered to indicate statistical significance. All statistical analyses were performed using the SAS software (SAS Institute, Cary, NC, USA version is 9.4).

## 3. Results

**Figure 3:**
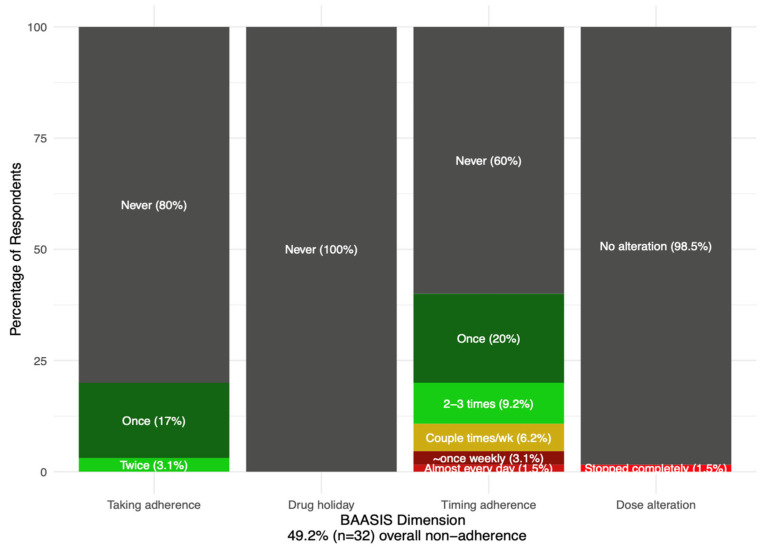
Adherence to immunosuppressants stratified by the taking adherence, drug holiday, timing adherence, and dose alteration dimensions.

As shown in Table 1, 49.2% of all participants reported nonadherence to immunosuppressant medication. No participants reported drug holidays; however, 40% took medication late, 20% missed at least one dose, and 40% had timing difficulties, with one participant reporting having difficulty taking their medication on time nearly every day. One participant stopped taking their medication entirely. Figure 3 illustrates these findings by BAASIS dimensions.

Participant characteristics were organized according to the five dimensions of adherence: sociodemographic, transplant, healthcare access, post-transplant treatment, and patient-related psychosocial factors. These characteristics are described for the entire sample and compared between adherent and non-adherent recipients (n = 65) in Table 2. Here we summarize participant characteristics overall across dimensions, and report key differences by adherence and organ transplant below.

Most of our participants were older White men (mean age 62 years; four years post-transplant) who were married, retired, and moderately educated, with 36% having completed a 4-year college degree and 76% earning high incomes of over USD 75,000. Most participants reported being satisfied with their financial situation and social life, having no difficulty performing daily physical activities, having good mental health, no adverse childhood experiences, and no community stress. All were non-smokers.

Half of the participants perceived unfair treatment (discrimination). The leading reason attributed to this discrimination was something other than the responses listed (32.3%), followed by race or skin color (19.4%). The most commonly reported coping response to discrimination was to accept it as a fact of life.

Significant differences between adherent and nonadherent recipients include that those who were nonadherent had fewer comorbidities of diabetes and obesity, poorer diet quality, and potentially more maladaptive coping responses to discrimination. For example, recipients in the nonadherent group reported that they expressed anger in response to discrimination more often than the adherent recipients.

The characteristics of participants in the heart and lung transplant groups were mostly similar. It should be noted that the heart transplant recipients had a higher number of Black recipients and more full-time employees. Separately, the lung transplant group had a greater proportion of female recipients. As such, when examining heart and lung transplant recipients separately (as shown in Table 3 and Table 4), patterns in differences between adherent and nonadherent participants mostly remained consistent with those observed in Table 2. However, they were not consistently statistically significant given the smaller sample sizes, and differences in perceived stress were observed.

Table 3 conveys the results obtained among the heart transplant recipients. The findings in the first three dimensions appear similar across the nonadherent and adherent groups. Aside from those findings in the fourth and fifth dimensions that aligned with those in the overall cohort, nonadherent heart recipients appeared to have more inadequate sleep and pain along with higher scores in perceived stress, discrimination in medical care, internalized racism, and depression.

In Table 4, patterns consistent with the overall cohort findings were again discovered when examining lung transplant recipients only. The variables in the first three dimensions appeared to be similar between the nonadherent and adherent groups. Different from the results in the fifth dimension found among heart transplant recipients, adherent and nonadherent lung recipients reported similar scores in terms of sleep quality, discrimination in medical care, pain, and depression, while nonadherent individuals reported slightly lower perceived stress and internalized racism scores.

## 4. Discussion

### 4.1. Overview

Guided by the five dimensions of adherence, the present study explored the factors associated with immunosuppressant medication adherence among a sample of thoracic transplant recipients enrolled in the GRAfT study, who were predominately White and at approximately four years post-transplantation. Half of the participants reported instances of medication nonadherence. No significant correlations were found within the first three dimensions of adherence, including sociodemographic factors (including race/ethnicity), transplant-related factors, and healthcare access-related factors. However, within the post-transplant-related dimension, adherent recipients exhibited significantly higher rates of comorbidities—particularly diabetes and obesity—when compared to their non-adherent counterparts. Corroborating our hypothesis, in the fifth patient-related psychosocial dimension, coping with discrimination—specifically, “expressing anger” and “working harder to prove others wrong”—showed a significant correlation with medication nonadherence. Based on our findings, barriers to immunosuppressant adherence in thoracic transplant recipients may include the timing and taking of medication, misperception of “good” health after transplant, and maladaptive coping responses to perceived discrimination.

### 4.2. Key Findings

Considering that we used self-reported measures, nonadherence in our sample of predominately White thoracic recipients was relatively high compared to existing studies. Utilizing the BAASIS scale, Denhaerynck and colleagues (2018) reported a nonadherence rate of 34.1%, while Zhang et al. (2019) reported a rate of 41.1% among heart transplant recipients [21,22,23,24]. Similarly, Drick and his team (2018) found a rate of 27.4% among lung transplant recipients [31]. Consistent with our findings, these studies identified two primary barriers to medication adherence: failing to take at least one dose on time (timing adherence) and skipping one or more consecutive doses (taking adherence) [21,24,31]. In a recent study involving predominantly Turkish lung transplant recipients, Bulbuloglu et al. (2024) reported that nearly 25% of their cohort presented irregular or complete discontinuation of immunosuppressive medications [65]. Moreover, evidence suggests that objective measures may reveal even higher rates of nonadherence. For example, when more objective methods were used to measure adherence—such as collateral provider reports, electronic monitoring, and blood assays—De Bleser and colleagues (2011) reported 60% and 72.2% nonadherence rates among heart and lung transplant recipients, respectively [66]. This implies that our subjective self-reported measures are likely conservative.

Perceived good health after transplantation may present a barrier to medication adherence in thoracic recipients [67,68]. Our findings indicate that individuals with fewer diet-related comorbidities, such as obesity and diabetes, along with poorer diet quality, tend to report higher nonadherence. Research shows that self-perception of good health can be significantly and negatively associated with treatment (medication and lifestyle) adherence among both recipients managing multiple comorbidities and those who are not [69,70]. In our study, individuals with fewer comorbid conditions may prioritize preventive health behaviors less than those who need to strictly adhere to medication and lifestyle regimens to manage their conditions. Supporting this hypothesis, poor diet quality was significantly more common in the nonadherent group. Given that positive health behaviors tend to co-occur, in addition to the significance of healthy dietary habits in lowering the risk of chronic disease, diet quality-related interventions could be an effective and strategic approach to increase medication adherence in thoracic transplant recipients [71,72]. This topic warrants further research, as there is little to no evidence beyond our data describing diet quality in this population.

In our study, we discovered that recipients who expressed anger and sought to prove others wrong as coping responses to perceived discrimination exhibited lower medication adherence. Although these behaviors can be classified as active coping strategies—often seen as positive mechanisms—long-term reliance on these strategies under stressful conditions has been linked to adverse health outcomes. Some evidence suggests that a tendency toward anger may increase the risk of negative health consequences, including cardiovascular disease (CVD), with White men being particularly vulnerable compared to Black men [73]. Furthermore, sustained high effort in coping with discrimination can lead to detrimental health effects due to mental and physical fatigue, which relates to the concept of John Henryism [74]. Initially developed to evaluate the negative health impacts of racism on Black men, this phenomenon has not been observed in White men, although evidence remains limited.

In a study predominantly involving White men, Allen et al. (2020) examined the mechanisms behind racial health disparities, focusing on the relationships between coping strategies and psychological and physiological stress response [75]. They found that both White and Black men frequently resort to maladaptive coping mechanisms when faced with psychological distress, such as denial, behavioral disengagement, stress eating, and smoking [75]. These findings align with our results, where the most common response to discrimination was to “accept it as a fact of life,” a passive coping mechanism akin to denial and behavioral disengagement. Moreover, withdrawing from treatment, like not taking medication or not maintaining essential preventative behaviors (e.g., healthy diet), can also be viewed as a form of behavioral disengagement [76].

Interestingly, while our sample reported experiences of discrimination, the primary reason for these perceptions were not fully captured in our study. While we considered factors such as race, gender, age, weight, height, education, income, physical disability, and religion, most respondents selected “other” to describe their experiences. This may indicate a need for further research into the experiences of White men and transplant recipients who perceive discrimination and how this impacts medication adherence and related health outcomes.

Taken together, potential barriers to adherence to immunosuppressant medications among thoracic transplant recipients include medication timing and taking, misconceptions about post-transplant health, and maladaptive coping with toxic stress such perceived discrimination. These issues highlight the need for further research, as they may affect nonadherence overall and contribute to racial and ethnic health disparities regarding long-term health outcomes in this population.

### 4.3. Limitations and Strengths

Our study highlights a significant gap in the literature regarding the prevalence of immunosuppressant adherence among thoracic transplant recipients and the social factors that may predict it; however, it has substantial limitations in terms of design, measurement, and generalizability. The cross-sectional design captures adherence behaviors at a single point in time, which prevents us from assessing causality. We interpret the findings as adherence factors that may act as barriers to medication adherence—our primary outcome. However, due to our cross-sectional study design, we cannot determine the direction of these associations. Including recipients from only one GRAfT site out of five resulted in a smaller sample size, which severely limited our statistical power to detect significant associations between the assessed factors and medication nonadherence. In our study, several factors previously reported in the literature as being significantly associated with nonadherence—such as public health insurance, income-related variables, and perceived social support—were not found to be significant [21,26,27,31,77]. Although our sample size was small, significant correlations were still detected, potentially suggesting that our estimates may be conservative.

Reliance on self-reported data lends our study to reporting bias and a lack of objective verification. As for medication adherence, more objective methods, such as drug-level monitoring, pill counts, or pharmacy refill records, would provide more verifiable data [17]. The self-reported comorbid conditions in our survey (obesity, diabetes history, and COVID-19) are also limited. A more robust assessment, involving objective measures such as the Charlson Comorbidity Index, could have provided a more comprehensive evaluation of participant comorbidities. Our study did not specify the types of medications prescribed to transplant recipients. This limitation may relate to the burden of care, including complex dosing regimens and side effects associated with the medication [17,20,78]. We also posed sensitive questions regarding social determinants of health and adherence to treatment, which may have resulted in social desirability bias [79].

Participants in the present study were drawn from the larger GRAfT study, which may have introduced sampling bias and limited the generalizability of our findings to the broader population of thoracic transplant recipients. Specifically, since the majority of our participants were older White men who were married, retired, moderately educated, and of middle income, this restricts the applicability of our findings to other racial and ethnic groups, as well as to individuals with both low and high incomes and high levels of education. Generating more evidence regarding barriers to medication adherence within these populations is potentially important for understanding the social disparities in adherence [80].

Our current objective is to gather preliminary data to support further studies involving more rigorous analyses. As this is the first time this survey has been conducted in this population, there were several limitations to our analysis due to the pilot nature of the survey and the small sample size. These data were collected from a single site of the parent study, Genomic Alliance for Transplantation (GRAfT), which included N = 497 heart (44%) and lung (56%) transplant recipients. The present single-site study is descriptive and does not include a power analysis. Our planned subsequent study will enroll 200 participants, including an additional site, to ensure adequate power for a multivariable analysis of racism, resilience, and adherence (effect size f^2^ = 0.04). Additionally, due to the small sample size characterizing the initial data, we will reserve Cronbach’s alpha calculations until we reach our planned enrollment of N = 200.

Despite these limitations, our study addresses several gaps in the literature. Among thoracic transplant recipients, this is the first study to our knowledge to assess the following specific factors within the patient-related psychosocial dimension of adherence: diet quality, medical mistrust, discrimination, adverse childhood experiences, and coping responses to discrimination. Our survey was administered by trained public health students. Since our participants were enrolled from those in the GRAfT study, we can follow up at subsequent sites for additional data, allowing for the prediction of long-term health outcomes with a larger sample size.

### 4.4. Public Health Implications

Our research identified barriers to immunosuppressant medication adherence among thoracic organ transplant recipients. This issue affects recipients, healthcare providers, researchers, and policymakers. Key factors influencing adherence include missed doses, inconsistent timing, and poor stress management. To improve adherence, it is essential to raise awareness, identify barriers, and incorporate medication reminders and stress management techniques—such as mindfulness-based stress reduction (MBSR) and meditation—into post-transplantation routines [33,81].

During follow-up visits, healthcare providers can engage in collaborative communication to discuss strategies for improved medication adherence, including stress management plans. This includes reviewing and simplifying complex medication schedules, especially for those with multiple chronic conditions [81]. In addition to physicians and nurses, consulting with a clinical pharmacist can also help to prevent miscommunication regarding taking and timing. Additionally, pharmacists can assist in identifying cost-effective brands and appropriate pill combinations, helping to reduce the perceived stress relating to medication adherence.

To further build research on health promotion in thoracic transplant recipients, researchers could conduct studies to evaluate technology-mediated interventions tailored for this population. These interventions might include low-cost reminder devices such as pill bottle strips, digital timer caps, or standard pillboxes, as well as more advanced options such as electronic packaging, AI algorithms, and ingestible biosensors [82,83,84]. There is limited evidence regarding the utility of these innovations for transplant recipients. Such studies could provide valuable insights for the development of effective strategies to improve medication adherence.

Healthcare facilities may provide health communications and resources related to the topics mentioned above. This process can involve additional training to address medication barriers that are not traditionally discussed during these appointments, such as perceived discrimination, coping responses, and diet quality. By doing so, providers may suggest wrap-around services related to stress management and wellbeing, allowing them to review medication adherence while also seeking training on cultural humility. This approach may help to prevent the patient from experiencing excess stress during medical visits.

As for policies, our findings may lead to new screening tools for the monitoring of nonadherence and health outcomes in post-transplant recipients. For example, a tool that combines objective and subjective measures of medication adherence, along with social determinants such as perceived stress, could provide a clearer risk profile for nonadherence [24,66]. Additionally, policies that improve access to healthcare services—such as expanding insurance coverage for the resources mentioned above—may enhance adherence rates [81]. Overall, our findings support a multilevel approach including collaborative decision-making between recipients and providers, as well as supportive healthcare systems, in order to improve medication adherence in thoracic transplant recipients.

## 5. Conclusions

Our study highlighted a significant gap in the literature by examining the prevalence of immunosuppressant nonadherence among thoracic transplant recipients. It explored correlates of nonadherence in five different dimensions and identified potential barriers. Although the presented findings are preliminary, they emphasize the importance of a multi-level, interdisciplinary approach to understand and improve medication nonadherence and achieve overall health promotion in transplant recipients.

Effective strategies may include focusing on often-overlooked factors, such as diet quality, managing comorbid conditions, and coping mechanisms related to perceived discrimination. Looking ahead, a larger sample size will enhance our ability to identify significant associations and improve the generalizability of our findings. It would also enable a more thorough investigation of racial and ethnic disparities in medication adherence and long-term health markers (e.g., cell-free DNA levels) which are critical for the survival of thoracic transplant recipients.

In summary, further research on barriers to medication adherence in thoracic transplant recipients will be important for the development of personalized interventions that promote effective post-transplant self-management, as well as informing healthcare policies aimed at improving post-transplant care services.

## Figures and Tables

**Table 1:** Adherence to immunosuppressants as measured by BAASIS questionnaire in the total sample of thoracic transplant recipients (n = 65).

Dimension	BAASIS Items	Response	N (%)
Taking adherence	Do you remember missing a dose of your anti-rejection medications in the last four weeks? How often?	Never	52 (80.0)
		Once	11 (16.9)
		Twice	2 (3.1)
		Three times	-
		Four times	-
		More than four times	-
Drug holiday	Do you remember having skipped two or more doses of your anti-rejection medications in a row in the last four weeks? How often?	Never	65 (100.0)
		Once	-
		Twice	-
		Three times	-
		Four times	-
		More than four times	-
Timing adherence	Do you remember having taken your anti-rejection medication more than 2 h before or after the recommended dosing time in the last four weeks? How often?	Never	39 (60.0)
		Once	13 (20.0)
		Two to three times	6 (9.2)
		About once weekly	2 (3.1)
		A couple times per week	4 (6.2)
		Almost every day	1 (5.4)
Dose alteration	Have you altered the prescription amount (e.g., taken more or fewer pills or changed your dose) of your anti-rejection medication during the last four weeks without your doctor telling you to do so? Have you stopped taking your anti-rejection medications completely within the last year without your doctor telling you to do so?	No	64 (98.5)
		Altered amount	-
		Stopped completely	1 (1.5)
Overall level of nonadherence to immunosuppressant medication			32 (49.2%)

**Table 2:** Description of sociodemographic, transplant-related, healthcare system access, post-transplant treatment-related, and patient-related psychosocial factors by overall adherence in the total sample of thoracic transplant recipients.

Characteristics	All Recipients N = 65	Adherent N = 33, 50.8%	Nonadherent N = 32, 49.2%	*p*
**Sociodemographic factors**				
Age, mean (SD)	62 (9)	63 (8)	62 (10)	0.74
Female, n (%)	22 (33.8)	12 (36.4)	10 (31.3)	0.66
Race/ethnicity, n (%)				
White	47 (72.3)	24 (72.7)	23 (71.9)	0.82
Black	14 (21.5)	8 (24.2)	6 (18.8)	
Hispanic	1 (1.5)	0 (0.0)	1 (3.1)	
Other	3 (4.6)	1 (3.0)	2 (6.3)	
Education, n (%)				
Highschool graduate	8 (12.3)	5 (15.2)	3 (9.4)	0.53
Some college	21 (32.3)	11 (33.3)	10 (31.3)	
4-year college degree	19 (29.2)	7 (21.2)	12 (37.5)	
Graduate school	17 (26.2)	10 (30.3)	7 (21.9)	
Married, n (%)	56 (86.2)	29 (87.9)	27 (84.4)	0.73
Employment, n (%)				
Full-time	17 (26.2)	8 (24.2)	9 (28.1)	0.78
Part-time	2 (3.1)	1 (3.0)	1 (3.1)	0.99
Self-employed	3 (4.6)	2 (6.1)	1 (3.1)	0.99
Unemployed	2 (3.1)	2 (6.1)	0 (0.0)	0.49
Retired	35 (53.8)	18 (54.5)	17 (53.1)	0.99
Student	1 (1.5)	0 (0.0)	1 (3.1)	0.49
Homemaker	5 (7.7)	2 (6.1)	3 (9.4)	0.67
Disabled	11 (16.9)	7 (21.2)	4 (12.5)	0.51
Annual income, n (%)				
<USD 35,000	5 (8.1)	3 (9.7)	2 (6.5)	0.99
USD 35,000–49,999	4 (6.5)	2 (6.5)	2 (6.5)	
USD 50,000–74,999	6 (9.7)	3 (9.7)	3 (9.7)	
≥USD 75,000	47 (75.8)	23 (74.2)	24 (77.4)	
**Transplant-related factors**				
Years after transplantation median (IQR)	4 (3–5)	4 (3–5)	4 (3–5)	0.99
Age at the time of transplantation in years, mean (SD)	59 (9)	59 (8)	58 (10)	0.61
**Healthcare system access factors**				
Medical mistrust, median (IQR) Higher score indicates greater medical mistrust (1–5)	1.5 (1.0–2.0)	1.3 (1.0–1.8)	1.8 (1.0–2.0)	0.15
**Post-transplant treatment-related factors**				
Comorbidities				
Obesity BMI ≥ 30, n (%) [n = 64]	13 (20.3)	10 (30.3)	3 (9.7)	0.04 *
Diabetes, n (%)	30 (46.2)	20 (60.6)	10 (31.3)	0.02 *
Ever diagnosed with COVID-19 n (%)	13 (20.0)	9 (27.3)	4 (12.5)	0.14
**Patient-related psychosocial factors**				
***Physical Health***				
Physical activity, median (IQR) ≥24: high 14–23: moderate <14: low	36 (21–48)	30 (16–41)	42 (26–52)	0.08
WELL Diet score, median (IQR) Range: 0–120 Higher score indicates better diet quality	73 (63–82)	77 (69–84)	70 (59–77)	0.04 *
Adequate sleep (7–9 h), n (%)	41 (63.1)	22 (66.7)	19 (59.4)	0.54
Smoking status, n (%)	0	0	0	0
***Mental Health***				
Perceived stress, median (IQR) Range: 0–16 A higher score indicated greater perceived stress	2.0 (1.0–5.0)	2.0 (1.0–5.0)	3.0 (1.0–5.5)	0.46
Everyday discrimination, median (IQR)				
Situation Range: 0–5 Higher scores reflect greater median number of types of discrimination events experienced	2 (0–3)	2 (1–3)	2 (0–3)	0.95
Frequency Range: 5–30 Higher scores reflect higher median number of discrimination events	7 (5–10)	7 (6–9)	7 (5–10)	0.89
Reason for Discrimination, n (%) [n = 31] Higher score corresponds to more participants				
Age	4 (12.9)	2 (14.3)	2 (11.8)	0.99
Gender	2 (6.5)	2 (14.3)	0 (0.0)	0.20
Race/skin color	6 (19.4)	2 (14.3)	4 (23.5)	0.66
Weight or height	3 (9.7)	1 (7.1)	2 (11.8)	0.99
Education or income	1 (3.2)	0 (0.0)	1 (5.9)	0.99
Physical disability	4 (12.9)	2 (14.3)	2 (11.8)	0.99
Religion	1 (3.2)	0 (0.0)	1 (5.9)	0.99
Other	10 (32.3)	5 (35.7)	5 (29.4)	0.99
Coping with Discrimination, n (%) Higher score corresponds to more participants				
Tried to do something about it	25 (38.5)	13 (39.4)	12 (37.5)	0.99
Accepted it as a fact of life	44 (67.7)	22 (66.7)	22 (68.8)	0.99
Worked harder to prove them wrong	24 (36.9)	8 (24.2)	16 (50.0)	0.03 *
Realized you brought it on yourself	10 (15.4)	3 (9.1)	7 (21.9)	0.15
Talked to someone about how you were feeling	28 (43.1)	11 (33.3)	17 (53.1)	0.11
Expressed anger or got mad	23 (35.4)	7 (21.2)	16 (50.0)	0.02 *
Prayed about the situation	25 (38.5)	11 (33.3)	14 (43.8)	0.39
Life discrimination events median (IQR)	1 (0–2)	1 (0–2)	0 (0–1)	0.14
Discrimination in medical care, n (%)	5 (7.7)	1 (3.0)	4 (12.5)	0.20
Internalized racism, median (IQR) [n = 63]	2.2 (1.6–2.6)	2.0 (1.6–2.6)	2.2 (1.6–2.6)	0.67
Community stress, median (IQR)	0 (0–1)	0 (0–0)	0 (0–1)	0.15
ACES, median (IQR)	0 (0–2)	0 (0–1)	0 (0–2)	0.67
Financial stress, median (IQR) Range = 1–5 Higher score reflects more financial stress	1.5 (1.0–2.0)	1.5 (1.0–2.0)	1.5 (1.0–2.0)	0.82
Global health ratings (PROMIS)				
Pain in past week, median (IQR) Range = 0–10 Higher score reflects higher pain level	2 (0–5)	2 (1–5)	2 (0–5)	0.34
Physical health, n (%)				
Poor/fair	13 (20.0)	7 (21.2)	6 (18.8)	0.83
Good	28 (43.1)	13 (39.4)	15 (46.9)	
Very good/excellent	24 (36.9)	13 (39.4)	11 (34.4)	
Mostly/completely able to carry out daily physical activities, n (%)	57 (87.7)	29 (87.9)	28 (87.5)	0.99
Very good/excellent mental health, n (%)	48 (73.8)	22 (66.7)	26 (81.3)	0.18
Very good/excellent satisfaction with social activities and relationships, n (%) [n = 64]	45 (70.3)	20 (60.6)	25 (80.6)	0.08
Social support level, n (%) [n = 64]				
Low	3 (4.7)	1 (3.0)	2 (6.5)	0.45
Moderate	42 (65.6)	20 (60.6)	22 (71.0)	
High	19 (29.7)	12 (36.4)	7 (22.6)	
Depression (PHQ-2), n (%) Range = 0–6 Scores of 2 or more indicate high likelihood of major depressive disorder				
0–1	53 (81.5)	28 (84.8)	25 (78.1)	0.80
2	8 (12.3)	3 (9.1)	5 (15.6)	
3–4	4 (6.2)	2 (6.1)	2 (6.3)	

* indicates *p* < 0.05.

**Table 3:** Description of sociodemographic, transplant-related, healthcare access, post-transplant treatment-related, and patient-related psychosocial factors by overall adherence in heart transplant recipients.

Characteristics	Overall N = 32	Adherent N = 17, 53.1%	Nonadherent N = 15, 46.9%
**Sociodemographic factors**			
Age, mean (SD)	64 (8)	65 (6)	62 (9)
Female, n (%)	7 (21.9)	3 (17.6)	4 (26.7)
Race/ethnicity, n (%)			
White	20 (62.5)	10 (58.8)	10 (66.7)
Black	11 (34.4)	7 (41.2)	4 (26.7)
Hispanic	0 (0.0)	0 (0.0)	0 (0.0)
Other	1 (3.1)	0 (0.0)	1 (6.7)
Education, n (%)			
High school graduate	4 (12.5)	3 (17.6)	1 (6.7)
Some college	10 (31.3)	6 (35.3)	4 (26.7)
4-year college degree	11 (34.4)	2 (11.8)	9 (60.0)
Graduate school	7 (21.9)	6 (35.3)	1 (6.7)
Married, n (%)	28 (87.5)	16 (94.1)	12 (80.0)
Employment, n (%)			
Full-time	11 (34.4)	5 (29.4)	6 (40.0)
Part-time	1 (3.1)	1 (5.9)	0 (0.0)
Self-employed	1 (3.1)	0 (0.0)	1 (6.7)
Unemployed	2 (6.3)	2 (11.8)	0 (0.0)
Retired	15 (46.9)	9 (52.9)	6 (40.0)
Student	1 (3.1)	0 (0.0)	1 (6.7)
Homemaker	2 (6.3)	0 (0.0)	2 (13.3)
Disabled	3 (9.4)	2 (11.8)	1 (6.7)
Annual income, n (%) [n = 31]			
<USD 35,000	2 (6.5)	2 (12.5)	0 (0.0)
USD 35,000–49,999	1 (3.2)	0 (0.0)	1 (6.7)
USD 50,000–74,999	3 (9.7)	2 (12.5)	1 (6.7)
≥USD 75,000	25 (80.6)	12 (75.0)	13 (86.7)
**Transplant-related factors**			
Years after transplantation median (IQR)	4 (3–5)	4 (3–5)	4 (3–5)
Age at the time of transplantation in years, mean (SD)	59 (7)	61 (6)	57 (9)
**Healthcare system access factors**			
Medical mistrust, median (IQR) A higher score indicates greater medical mistrust (1–5)	1.8 (1.0–2.0)	1.3 (1.0–2.0)	1.8 (1.7–2.0)
**Post-transplant treatment-related factors**			
Comorbidities			
Obesity BMI ≥ 30, n (%) [n = 64]	7 (21.9)	6 (35.3)	1 (6.7)
Diabetes, n (%)	16 (50.0)	12 (70.6)	4 (26.7)
Ever diagnosed with COVID-19 n (%)	8 (25.0)	6 (35.3)	2 (13.3)
**Patient-related psychosocial factors**			
***Physical Health***			
Physical activity, median (IQR) ≥24: high 14–23: moderate < 14: low	30 (20–56)	21 (16–48)	35 (21–56)
WELL Diet score, median (IQR) Range: 0–120 Higher score indicates better diet quality	73 (63–79)	76 (68–82)	71 (57–76)
Sleep quality, n (%)	15 (46.9)	9 (52.9)	6 (40.0)
Smoking status, n (%)	0	0	0
***Mental Health***			
Perceived stress, median (IQR) Range: 0–16 A higher score indicates greater perceived stress	2.5 (1.0–5.0)	2.0 (1.0–5.0)	3.0 (1.0–5.0)
Everyday discrimination, median (IQR)			
Situation Range: 0–5 Higher scores reflect greater median number of types of discrimination events experienced	2 (1–3)	2 (1–3)	2 (1–3)
Frequency Range: 5–30 Higher scores reflect higher median number of discrimination events	8 (6–11)	8 (6–11)	8 (6–10)
Chronicity Range: Higher scores reflect higher median number of discrimination events experienced annually	3 (1–8)	3 (1–7)	3 (1–10)
Reason for Discrimination, n (%) [n = 18] Higher score corresponds to more participants			
Age	3 (16.7)	2 (22.2)	1 (11.1)
Gender	1 (5.6)	1 (11.1)	0 (0.0)
Race/skin color	4 (22.2)	2 (22.2)	2 (22.2)
Weight or height	2 (11.1)	0 (0.0)	2 (22.2)
Education or income	0 (0.0)	0 (0.0)	0 (0.0)
Physical disability	1 (5.6)	0 (0.0)	1 (11.1)
Religion	0 (0.0)	0 (0.0)	0 (0.0)
Other	7 (38.9)	4 (44.4)	3 (33.3)
Coping with Discrimination, n (%) Higher score corresponds to more participants			
Tried to do something about it	13 (40.6)	8 (47.1)	5 (33.3)
Accepted it as a fact of life	25 (78.1)	13 (76.5)	12 (80.0)
Worked harder to prove them wrong	12 (37.5)	5 (29.4)	7 (46.7)
Realized you brought it on yourself	4 (12.5)	1 (5.9)	3 (20.0)
Talked to someone about how you were feeling	12 (37.5)	4 (23.5)	8 (53.3)
Expressed anger or got mad	14 (43.8)	5 (29.4)	9 (60.0)
Prayed about the situation	15 (46.9)	6 (35.3)	9 (60.0)
Life discrimination events median (IQR)	1 (0–3)	2 (0–3)	0 (0–3)
Discrimination in medical care, n (%)	3 (9.4)	0 (0.0)	3 (20.0)
Internalized racism, median (IQR) [n = 31]	2.0 (1.4–2.6)	1.8 (1.4–2.2)	2.3 (1.8–2.8)
Community stress, median (IQR)	0 (0–0)	0 (0–0)	0 (0–0)
ACES, median (IQR)	0 (0–2)	0 (0–1)	0 (0–2)
Financial stress, median (IQR) Range = 1–5 Higher score reflects more financial stress	1.5 (1.0–2.0)	1.5 (1.0–2.0)	1.5 (1.0–2.0)
Global health ratings (PROMIS)			
Pain in past week, median (IQR) Range = 0–10 Higher score reflects higher pain level	2 (1–5)	2 (2–5)	1 (0–5)
Physical health, n (%)			
Poor/fair	5 (15.6)	3 (17.6)	2 (13.3)
Good	12 (37.5)	7 (41.2)	5 (33.3)
Very good/excellent	15 (46.9)	7 (41.2)	8 (53.3)
Mostly/completely able to carry out daily physical activities, n (%)	26 (81.3)	14 (82.4)	12 (80.0)
Very good/excellent mental health, n (%)	23 (71.9)	11 (64.7)	12 (80.0)
Very good/excellent satisfaction with social activities and relationships, n (%) [n = 64]	23 (74.2)	11 (64.7)	12 (85.7)
Social support level, n (%) [n = 31]			
Low	2 (6.5)	1 (5.9)	1 (7.1)
Moderate	17 (54.8)	8 (47.1)	9 (64.3)
High	12 (38.7)	8 (47.1)	4 (28.6)
Depression (PHQ-2), n (%) Range = 0–6 Scores of 2 or more indicate high likelihood of major depressive disorder			
0–1	26 (81.3)	15 (88.2)	11 (73.3)
2	5 (15.6)	2 (11.8)	3 (20.0)
3–4	1 (3.1)	0 (0.0)	1 (6.7)

**Table 4:** Description of sociodemographic, transplant-related, healthcare system access, post-transplant treatment-related, and patient-related psychosocial factors by overall adherence in lung transplant recipients.

Characteristics	Overall N = 33	Adherent N = 16, 53.1%	Nonadherent N = 17, 46.9%
**Sociodemographic factors**			
Age, mean (SD)	61 (10)	61 (10)	62 (10)
Female, n (%)	15 (45.5)	9 (56.3)	6 (35.3)
Race/ethnicity, n (%)			
White	27 (81.8)	14 (87.5)	13 (76.5)
Black	3 (9.1)	1 (6.3)	2 (11.8)
	1 (3.0)	0 (0.0)	1 (5.9)
Other	2 (6.1)	1 (6.3)	1 (5.9)
Education, n (%)			
High school graduate	4 (12.1)	2 (12.5)	2 (11.8)
Some college	11 (33.3)	5 (31.3)	6 (35.3)
4-year college degree	8 (24.2)	5 (31.3)	3 (17.6)
Graduate school	10 (30.3)	4 (25.0)	6 (35.3)
Married, n (%)	28 (84.8)	13 (81.3)	15 (88.2)
Employment, n (%)			
Full-time	6 (18.2)	3 (18.8)	3 (17.6)
Part-time	1 (3.0)	0 (0.0)	1 (5.9)
Self-employed	2 (6.1)	2 (12.5)	0 (0.0)
Unemployed	0 (0.0)	0 (0.0)	0 (0.0)
Retired	20 (60.6)	9 (56.3)	11 (64.7)
Student	0 (0.0)	0 (0.0)	0 (0.0)
Homemaker	3 (9.1)	2 (12.5)	1 (5.9)
Disabled	8 (24.2)	5 (31.3)	3 (17.6)
Annual income, n (%) [n = 31]			
<USD 35,000	3 (9.7)	1 (6.7)	2 (12.5)
USD 35,000–49,999	3 (9.7)	2 (13.3)	1 (6.3)
USD 50,000–74,999	3 (9.7)	1 (6.7)	2 (12.5)
≥USD 75,000	22 (71.0)	11 (73.3)	11 (68.8)
**Transplant-related factors**			
Years after transplantation median (IQR)	3 (2–5)	4 (3–5)	3 (2–5)
Age at the time of transplantation in years, mean (SD)	58 (10)	57 (10)	59 (11)
**Healthcare system access factors**			
Medical mistrust, median (IQR) A higher score indicates greater medical mistrust (1–5)	1.3 (1.0–2.0)	1.3 (1.0–1.8)	1.3 (1.0–2.0)
**Post-transplant treatment-related factors**			
Comorbidities			
Obesity BMI ≥ 30, n (%) [n = 64]	6 (18.8)	4 (25.0)	2 (12.5)
Diabetes, n (%)	14 (42.4)	8 (50.0)	6 (35.3)
Ever diagnosed with COVID-19 n (%)	5 (15.2)	3 (18.8)	2 (11.8)
**Patient-related psychosocial factors**			
***Physical Health***			
Physical activity, median (IQR) >24: high 14–23: moderate <14: low	36 (25–42)	33 (20–40)	42 (27–48)
WELL Diet score, median (IQR) Range: 0–120 Higher score indicates better diet quality	73 (66–84)	78 (71–87)	68 (59–81)
Sleep quality, n (%)	26 (78.8)	13 (81.3)	13 (76.5)
Smoking status, n (%)	0	0	0
***Mental Health***			
Perceived stress, median (IQR) Range: 0–16 A higher score indicates greater perceived stress	2.0 (1.0–6.0)	2.5 (1.0–6.0)	2.0 (1.0–6.0)
Everyday discrimination, median (IQR)			
Situation Range: 0–5 Higher scores reflect greater median number of types of discrimination events experienced	1 (0–3)	1 (0–2)	1 (0–3)
Frequency Range: 5–30 Higher scores reflect higher median number of discrimination events	7 (5–9)	6 (5–9)	7 (5–9)
Chronicity Range: Higher scores reflect higher median number of discrimination events experienced annually	1 (0–4)	1 (0–4)	2 (0–4)
Reason for Discrimination, n (%) [n = 13] Higher score corresponds to more participants			
Age	1 (7.7)	0 (0.0)	1 (12.5)
Gender	1 (7.7)	1 (20.0)	0 (0.0)
Race/skin color	2 (15.4)	0 (0.0)	2 (25.0)
Weight or height	1 (7.7)	1 (20.0)	0 (0.0)
Education or income	1 (7.7)	0 (0.0)	1 (12.5)
Physical disability	3 (23.1)	2 (40.0)	1 (12.5)
Religion	1 (7.7)	0 (0.0)	1 (12.5)
Other	3 (23.1)	1 (20.0)	2 (25.0)
Coping with Discrimination, n (%) Higher score corresponds to more participants			
Tried to do something about it	12 (36.4)	5 (31.3)	7 (41.2)
Accepted it as a fact of life	19 (57.6)	9 (56.3)	10 (58.8)
Worked harder to prove them wrong	12 (36.4)	3 (18.8)	9 (52.9)
Realized you brought it on yourself	6 (18.2)	2 (12.5)	4 (23.5)
Talked to someone about how you were feeling	16 (48.5)	7 (43.8)	9 (52.9)
Expressed anger or got mad	9 (27.3)	2 (12.5)	7 (41.2)
Prayed about the situation	10 (30.3)	5 (31.3)	5 (29.4)
Life discrimination events median (IQR)	0 (0–2)	1 (0–2)	0 (0–1)
Discrimination in medical care, n (%)	2 (6.1)	1 (6.3)	1 (5.9)
Internalized racism, median (IQR) [n = 32]	2.3 (1.6–2.6)	2.5 (1.7–2.8)	1.9 (1.5–2.4)
Community stress, median (IQR)	0 (0–1)	0 (0–0)	1 (0–1)
ACES, median (IQR)	0 (0–2)	0 (0–2)	0 (0–2)
Financial stress, median (IQR) Range = 1–5 Higher score reflects more financial stress	1.5 (1.0–2.0)	1.5 (1.0–2.0)	1.5 (1.0–2.0)
Global health ratings (PROMIS)			
Pain in past week, median (IQR) Range = 0–10 Higher score reflects higher pain level	3 (0–4)	2 (1–5)	3 (0–4)
Physical health, n (%)			
Poor/fair	8 (24.2)	4 (25.0)	4 (23.5)
Good	16 (48.5)	6 (37.5)	10 (58.8)
Very good/excellent	9 (27.3)	6 (37.5)	3 (17.6)
Mostly/completely able to carry out daily physical activities, n (%)	31 (93.9)	15 (93.8)	16 (94.1)
Very good/excellent mental health, n (%)	25 (75.8)	11 (68.8)	14 (82.4)
Very good/excellent satisfaction with social activities and relationships, n (%) [n = 64]	22 (66.7)	9 (56.3)	13 (76.5)
Social support level, n (%) [n = 64]			
Low	1 (3.0)	0 (0.0)	1 (5.9)
Moderate	25 (75.8)	12 (75.0)	13 (76.5)
High	7 (21.2)	4 (25.0)	3 (17.6)
Depression (PHQ-2), n (%) Range = 0–6 Scores of 2 or more indicate high likelihood of major depressive disorder			
0–1	27 (81.8)	13 (81.3)	14 (82.4)
2	3 (9.1)	1 (6.3)	2 (11.8)
3–4	3 (9.1)	2 (12.5)	1 (5.9)

## Data Availability

No new data were created or analyzed in this study. Data sharing is not applicable to this article.

## Abbreviations

The following abbreviations are used in this manuscript:

ACE	Adverse Childhood Experiences
BAASIS	Basel Assessment of Adherence with Immunosuppressive Medication Scale
BRS	Brief Resilience Scale
CDS	Coping with Discrimination
COVID-19	Coronavirus disease 2019
dd-cfDNA	Donor-derived cell-free DNA
EDS	Everyday Discrimination Scale
GRAfT	Genomic Research Alliance for Transplantation
GBMMS	Group-Based Medical Mistrust
IRS	Internalized Racism Scale
LUC	Loyola University Chicago
MED	Major Experiences of Discrimination
PHQ-2	Patient Health Questionnaire-2
PROMIS	Patient-Reported Outcomes Measurement Information System
PPS-4	Perceived Stress Scale
SIMSS	Single Item Measure of Social Support
WELL	Wellness Living Laboratory

## Appendix A

**Table A1:** Detailed description of study variables.

Dimension 1: Sociodemographic Factors	
Age	Self-reported birth date (e.g., mm/dd/yyyy). Open-ended.
Sex	Self-reported as male or female.
Race/ethnicity	Self-reported race/ethnicity. Response options were White, Black or African American, Hispanic or Latinx, and Other.
Education level	Self-reported highest level of education. Response options were less than a high school diploma, high school diploma or GED, some college or technical school, 4-year college degree, postgraduate degree.
Marital status	Self-reported marital status. Response options were (1 “single”, 2 “married”, 3 “living as married”, or 4 “widowed”).
Employment status	Self-reported employment status. Response options were employed full-time (FT), employed part-time (PT), self-employed, unemployed, retired, student, homemaker, or disabled.
Annual household income	Self-reported level of household income. Response options were Less than USD 35,000; USD 35,000 to 49,999; USD 50,000 to 74,999; and USD 75,000+.
**Dimension 2: Transplant-Related Factors**	
Years after transplantation	Calculated based on self-reported time of transplantation. Median and interquartile range were calculated.
Age at the time of transplantation	Measured based on self-reported age at transplantation. Mean and standard deviation were calculated.
**Dimension 3: Healthcare Access-Related Factors**	
Medical mistrust	The Group-Based Medical Mistrust (GBMMS) is a 12-item scale developed to measure race-based medical mistrust: the suspicion of mainstream healthcare systems and professionals and the treatment provided to individuals of the respondent’s ethnic or racial group [41]. While there is no existing literature that utilizes GBMMS among transplant recipients, the GBMMS has been used in other patient populations with chronic illness and has convergent validity demonstrated in studies that use GBMSS and other mistrust scales [85]. We asked participants six items related to GBMMS. Response options ranged from 1 to 5 (strongly disagree to strongly agree) and the mean of the responses was calculated to be the final score, which can range from 1–5. Higher scores indicate greater medical mistrust. Example: “People of my ethnic group cannot trust doctors and healthcare workers”.
**Dimension 4: Post-Transplant Treatment-Related Factors**	
Comorbidities	Self-reported weight and height, history of diabetes, and COVID-19 diagnosis. Weight and height data were used to calculate BMI and describe obesity (BMI ≥ 30).
**Dimension 5: Patient-Related Psychosocial Factors**	
Physical Health Outcomes	
Diet quality	Diet quality was measured by the WELL Diet Score, which has been correlated with the Alternative Healthy Eating Index-2010), a well-established measure of diet quality [42]. For the 12 diet-related items, the team of nutrition professionals working on the project agreed, by consensus, how to distribute points across the different frequencies of consumption. Participants were asked about their frequency of consumption. Example: “How often do you eat fish?”. Points were then combined to generate a total WELL diet quality score, which can range from 0 (indicating low quality diet) to 120 (indicating better diet quality).
Physical activity	Godin Leisure-Time Exercise Questionnaire has been employed across various populations, including healthy adults, individuals with chronic conditions, and cancer survivors to measure physical activity [43,86]. Participants were asked during a typical seven-day period (a week), how many times on average do you do the following kinds of exercise? Three items assessed the frequency per week of strenuous (heart beats rapidly), moderate (not exhausting) and mild (minimal effort) exercise for more than 15 min in addition to frequency of leisure and sedentary activity per week. One item assessed how often do you engage in regular activity long enough to work up a sweat. Response options were (1 “often”, 2 “sometimes”, and 3 “never/rarely”) [43]. Scores of 24 or above are considered a “high” level of physical activity, scores between 14 and 23 are considered “moderate” activity, and ones below 14 suggest a “low” level of physical activity.
Sleep quality	Self-reported hours of sleep per night. Participants were asked how many hours they slept per night during the past four weeks. Response options were (1 “5 h or less”, 2 “6 h”, 3 “7 h”, 4 “8 h”, 5 “9 h”, and 6 “10 or more hours”). Those with good sleep scores (7–9 h) are reported.
Smoking status	Self-reported smoking status. Do you smoke cigarettes now? (1 “yes”; 2 “no”).
Mental Health Outcomes	
General stress	The Perceived Stress Scale-4 (PSS-4) was used to measure the degree to which respondents find their lives unpredictable, uncontrollable, and overloaded [44]. The PSS-4 includes four items that measure self-appraised stress over the previous month. It has been found to be psychometrically valid in samples of African American women and has shown negative associations with overall health status and well-being. Response options ranged from “never” to “very often”.
Everyday Discrimination	Racial discrimination was assessed using five items from the Everyday Discrimination Scale (EDS) and was coded using two conventional approaches: (1) ‘situation-based coding’: number of different situations ever experienced; (2) ‘frequency-based coding’: sum of Likert scale responses ranging from never to almost every day; and (3) a new ‘chronicity-based coding’ approach: sum of responses, weighted to capture annual chronicity (e.g., ‘a few times a month’ = 3 × 12 = 36 ×/year) [49,53,87,88]. The EDS asks, “In your day-to-day life, how often do any of the following things happen to you?” and can include items such as, “You are treated with less courtesy or respect than other people”. Number of situations was reported, with response options ranged from never to almost every day. Higher scores indicate more types of situations where discrimination was experienced, greater frequency of discrimination, and greater chronicity.
Perceived Reasons for Discrimination	In the EDS, if participants answered “a few times a year” or more frequently to at least one everyday discrimination question, they were asked a follow-up question about the main reason for these experiences, with response options including race, gender, age, weight or height, education or income, physical disability, religion, or other. The frequency of the responses for each perceived reason were reported.
Coping Response to Unfair Treatment	Follow-up questions based on a version of the MED scale used in previous studies [46,47,48] asked participants eight items regarding their responses to unfair treatment. These response types included “tried to do something about it”, “accepted it as a fact of life”, “worked harder to prove them wrong”, “realized you brought it on yourself”, “talked to someone about how you were feeling”, “expressed anger or got mad”, and “prayed about the situation”. Response options were “yes” or “no”. The frequency of the responses for each coping response were reported.
Life Discrimination Events	Six items from the Major Experiences of Discrimination (Abbreviated Version) [53,87,88,89] were used to assess amount of lifetime discrimination events participants experienced. The MED asked participants, “Have any of the following ever happened to you?” Examples include “At any time in your life, have you ever been unfairly fired?” and “Have you ever been unfairly stopped, searched, questioned, physically threatened, or abused by the police?” Response options were “yes” or “no”. The frequencies of events were reported for each recipient.
Discrimination in Medical Care	A single item from the Major Experiences of Discrimination scale—based on an instrument used in the Coronary Artery Risk Development in Young Adults (CARDIA) study—asked participants: “Have you ever experienced discrimination, been prevented from doing something, or been hassled or made to feel inferior while getting medical care because of your race, ethnicity, or color?” Responses were “yes” or “no”. If yes, participants were asked how many times (once, two or three times, four or more times) [51].
Internalized racism	The Internalized Racism Scale (originally with 36 items) assesses internalized racism, referring to the acceptance of societal beliefs about one’s group, which can negatively affect self-esteem, mental health, and behavior [52]. The scale was abbreviated to assess five items related to belief biases, alteration of physical appearance, internalized negative stereotypes, hair change, and African worldview and motifs. Response options ranged from 1 (strongly disagree) to 5 (strongly agree). An example item is “There are no institutions of higher learning in Africa”. Scores range from 1 to 5, with higher scores indicating greater levels of internalized racial oppression.
Community stress	Community stress was measured using a five-item subscale adapted from the Project on Human Development in Chicago Neighborhoods, focusing on violence occurring in the community [53]. Participants were asked, “In the past 6 months, has there been (1) a neighborhood fight involving a weapon; (2) a violent argument between neighbors; (3) gang fights; (4) sexual assault or rape; (5) robbery or mugging?” Response options ranged from 1 (never) to 5 (very often). Scores range from 0–5. Higher score corresponded to higher community stress.
Early stress	The Adverse Childhood Experiences scale (ACES) from Felitti et al.’s original scale was used to measure stress and trauma experienced in the first 18 years of life [54]. Higher scores have been causally linked to mental illnesses, addictions, chronic disease, multi-organ medical diseases, and worsened quality-of-life [55,90,91]. Participants responded to eight items, with response options “yes”, “no”, or “refuse/don’t know”. An example item is “Physical abuse: How often did a parent or adult in your home ever hit, beat, kick, or physically hurt you in any way?”
Financial stress	Two routinely used questions on household financial situation and monthly financial strain were used to measure financial stress, which has been linked with psychological distress [56]. Participants were asked “How satisfied are you with your family’s financial situation?” with response options ranging from 1 (completely) to 5 (not at all), and “How difficult is it to meet monthly bill payments?” with response options ranging from 1 (not at all) to 5 (extremely). Scores range from 1–5. Higher scores correspond to greater financial stress.
Resilience	The Brief Resilience Scale (BRS), developed by Smith and colleagues in 2008, measures participants’ ability to bounce back from adversity [57]. Six items were used to assess the degree to which respondents agreed or disagreed with statements such as “I tend to bounce back quickly after hard times”. Response options ranged from “strongly agree” (1) to “strongly disagree” (5). Higher scores indicate greater resilience.
Global Health	Three items assessing global physical health and two items assessing global mental health derived from the Patient-Reported Outcomes Measurement Information System (PROMIS) Global Health (v 1.2) instrument were used to measure global health functioning. In kidney transplant recipients, the PROMIS-29 and PROMIS-57 (including the global health domain) showed strong internal consistency, structural validity, and test-retest reliability, supporting their use for assessing health-related quality of life in solid organ transplant recipients [92]. Participants were questioned regarding their physical health, ability to carry out daily physical activities, pain, mental health, and social satisfaction. Examples of questions include: “In general, how would you rate your physical health? In general, would you say your quality of life is: Excellent, Very Good, Good, Fair, Poor”. Pain was assessed with the question: “In the past 7 days, how would you rate your pain on average?” using a scale from 0 (no pain) to 10 (worst pain imaginable). Variations of the PROMIS Global Health scale have shown adequate reliability for group comparisons, and their associations with other indicators of health are similar to that of the original four-item scale [58,59].
Social support	The Single Item Measure of Social Support (SIMSS) [60] asked participants “How many people do you have near you that you can readily count on for help in times of difficulty, such as watching over children or pets, giving rides to the hospital or store, or helping when you are sick?” Response options were 0, 1, 2–5, 6–10, or more than 10. Responses of 0 or 1 indicated low tangible assistance, while 2–5 indicated moderate, and 6–10 or more indicated high tangible assistance. Although extremely short, this instrument is a strong predictor of morbidity and has good psychometric properties [60,93]. Use of this short measure allowed us to avoid utilizing one of the much longer questionnaires on social support, thus rendering the assessment process less burdensome for participants.
Depression	The Patient Health Questionnaire (PHQ-2) [61] is routinely used to screen patients for depression and asks participants two items regarding anhedonia and low mood and frequency over two weeks. It has been recommended by the American Heart Association Science Advisory for routinely screening cardiac patients for depression, and has been validated among solid organ transplant recipients [62,63,94]. Participants were asked, “Over the last 2 weeks, how often have you been bothered by the following problems?” Item 1 was “Little interest or pleasure in doing things”, and item 2 was “Feeling down, depressed, or hopeless”. Scores can range from 0–6 with four possible answer choices, starting with “not at all” (scored as 0) to “nearly every day (scored as 3)”. The final score is the sum of responses. Scores greater than or equal to 2 suggest the likelihood of major depressive disorder [64].
